# The Cascade Effect of Parent Dysfunction: An Emotion Socialization Transmission Framework

**DOI:** 10.3389/fpsyg.2020.579519

**Published:** 2020-10-15

**Authors:** Jessica A. Seddon, Rita Abdel-Baki, Sarah Feige, Kristel Thomassin

**Affiliations:** Department of Psychology, University of Guelph, Guelph, ON, Canada

**Keywords:** emotion, psychopathology, emotion regulation, parental emotion socialization, parent-child relations

## Abstract

The current study tested a preliminary cascade model of parent dysfunction—i.e., internalizing psychopathology and emotion dysregulation—whereby parent dysfunction is transmitted to children through the impact of parental emotion socialization on child emotion regulation. Participants were 705 mothers (*M*_age_ = 36.17, *SD* = 7.55) and fathers (*M*_age_ = 35.43, *SD* = 6.49) of children aged 8 to 12 years who self-reported on their internalizing psychopathology, emotion regulation difficulties, and emotion socialization practices, and on their child’s internalizing psychopathology and emotion regulation. Using a split sample method, we employed a data-driven approach to develop a conceptual model from our initially proposed theoretical model with the first subsample (*n* = 352, 51% mothers), and then validated this model in a second subsample (*n* = 353, 49% mothers). Results supported a model in which the transmission of dysfunction from parent to child was sequentially mediated by unsupportive parental emotion socialization—but *not* supportive parental emotion socialization—and child emotion dysregulation. The indirect effects from the final model did not differ by parent gender. Findings provide preliminary support for a mechanism by which maternal and paternal internalizing psychopathology *and* emotion dysregulation disrupt parental emotion socialization by increasing unsupportive emotion socialization practices, which impacts children’s development of emotion regulation skills and risk for internalizing psychopathology.

## Introduction

Parent internalizing psychopathology and emotion dysregulation (herein also referred to as “dysfunction”) are well-established risk factors for child psychopathology (e.g., Goodman and Brumley, 1990; Lapalme et al., 1997; DelBello and Geller, 2001; Buckholdt et al., 2014). Parent dysfunction may be transmitted to children through heritability (Rutter et al., 1999a; Hawn et al., 2015), environmental pathways (e.g., poor parenting, stress; Denham et al., 1997; Eisenberg et al., 1998; McLeod et al., 2007), and complex biology-by-environment interactions (Moffitt et al., 2006; Rutter et al., 2006). In particular, high levels of unsupportive parental emotion socialization (e.g., punitive and minimizing responses to children’s emotion), low levels of supportive parental emotion socialization (e.g., validation and encouragement of children’s emotion), and emotion regulation deficits have received special attention as factors contributing to the transmission of dysfunction from parent to child. Although several mediators have been identified and examined independently, an investigation of how these factors function in tandem has only briefly been explored (e.g., Suveg et al., 2011; Kerns et al., 2017; Thomassin et al., 2017). This is particularly important to examine given that these factors likely interact in complex ways in the transmission of dysfunction. In the current study, we conducted a preliminary test of a cascade model accounting for the transmission of parent internalizing psychopathology and emotion dysregulation to children via the impact of emotion socialization on child emotion dysregulation.

### Emotion Regulation

A specific factor by which parent dysfunction may be transmitted to children is through deficits in emotion regulation, as the inability to regulate emotions has been related to dysfunction and psychopathology in both children and adults (e.g., Bradley et al., 2011; Hofmann et al., 2012). Emotion regulation is defined as one’s ability to manage or adjust their emotional experiences to an appropriate level of intensity in order to accomplish their goals (Eisenberg and Spinrad, 2004; Thompson and Goodvin, 2007). Children’s adaptive emotion regulation skills have been associated with positive socioemotional outcomes (Maughan et al., 2007; Blair et al., 2014; Thomassin et al., 2017; Harrington et al., 2020), including academic success and friendship quality. On the other hand, maladaptive emotion regulation strategies and emotion dysregulation have been identified as transdiagnostic risk factors for internalizing psychopathology (Southam-Gerow and Kendall, 2000; Abela et al., 2002; Silk et al., 2003; Suveg and Zeman, 2004; Hofmann et al., 2012). For example, in a cross-sectional group of children in the seventh and 10th grades, Silk et al. (2003) found that the use of less effective emotion regulation strategies was associated with increased symptoms of depression and problem behaviors. In another cross-sectional study, Suveg and Zeman (2004) similarly found that children aged 8 to 12 years old with anxiety disorders were more likely than those without anxiety disorders to experience dysregulation around feelings of worry, sadness, and anger. Children with anxiety disorders were also less able to cope with these feelings, and experience parent-reported emotion regulation difficulties (Suveg and Zeman, 2004). These findings illustrate the importance of examining the factors that contribute to children’s development of emotion regulation strategies.

### Associations Between Parent Dysfunction, Parental Emotion Socialization, and Child Emotion Regulation

Parental emotion socialization is one parent factor that has received some attention as a contributor to children’s development of emotion regulation skills given its established influence on children’s understanding and awareness of emotion (Eisenberg and Fabes, 1994; Eisenberg et al., 1998). Parental emotion socialization is the process by which parents teach their children about emotions and how they should be expressed and managed (Eisenberg et al., 1998; Morris et al., 2007). Parents may socialize their children in overt and direct ways through discussion about emotions, or in more subtle and indirect ways, such as through their reactions or responses to children’s displays of emotion and by modeling their own emotion-related expressions (Eisenberg et al., 1998; Chaplin et al., 2005). While several emotion socialization practices exist, they can be subsumed under two broadband approaches—supportive and unsupportive emotion socialization (i.e., Eisenberg et al., 1992, 1996; Eisenberg and Fabes, 1994; Gottman et al., 1997). Supportive emotion socialization includes practices such as validating and encouraging emotion expression, coaching children through emotional experiences, and modeling adaptive emotion regulation strategies (Gottman et al., 1997). These practices help children develop skills to effectively regulate and navigate their emotional experiences (Gottman et al., 1997), and have been found to be associated with greater social competence, coping, life satisfaction, and emotion regulation abilities (Roberts and Strayer, 1987; Yap et al., 2008; Gentzler et al., 2015; Ramakrishnan et al., 2019). For example, in one longitudinal study, experiencing maternal supportive reactions to negative emotions when 5-years-old was associated with better emotion regulation at age 10, and overall adjustment at age 15 (Perry et al., 2020). In contrast, unsupportive emotion socialization occurs when parents dismiss, minimize, or express distress toward their child’s emotion expression. Parents might teach children that the expression of certain emotions is inappropriate through repeated minimization and punitive responses to children’s emotion, which in turn can lead to poor child emotion regulation skills (Eisenberg et al., 1992, 1996; Eisenberg and Fabes, 1994). For example, a cross-sectional study by Sanders et al. (2015) found that children aged 8 to 11 years who perceived their parents as dismissive of their emotional experiences reported more emotion regulation difficulties than children who perceived their parents as supportive. Taken together, the literature indicates that supportive and unsupportive parental emotion socialization practices impact children’s development of emotion regulation abilities.

Effective parental emotion socialization is likely to be hindered by parent dysfunction, suggesting that it may play in a role in the transmission of dysfunction from parent to child. More specifically, parent internalizing psychopathology and emotion dysregulation have been shown to adversely affect parents’ ability to effectively respond to their children’s emotions (e.g., Brenner and Salovey, 1997; Eisenberg et al., 1998; Cao et al., 2017; Lovejoy et al., 2000; discussed in Havighurst and Kehoe, 2017). Researchers have found that symptoms of depression in mothers interfere with their ability to scaffold their child’s emotion regulation skill development (Goodman and Gotlib, 1999; Hoffman et al., 2006). Depressed mothers may not be emotionally available (Brenner and Salovey, 1997) nor sensitive to their child’s needs (Leadbeater et al., 1996; Donovan et al., 1998), and therefore may have difficulty tuning into and supporting their child’s emotional needs. Indeed, Field et al. (1990) found that mothers with depression were less likely to synchronize with the emotion expression of their 3-month old infants during play in a cross-sectional study. Parents with general emotion regulation difficulties are also less likely to effectively model and teach adaptive emotion regulation strategies to their children (see Morris et al., 2007, for a review). Indeed, a parent’s ability to regulate their own emotions influences how they socialize their child’s emotionality which communicates important messages about the appropriateness of their child’s emotion expression (Fabes et al., 2001; Zeman et al., 2006). In a cross-sectional study by Bariola et al. (2012), older children and adolescents aged 9 to 19 years were more likely to use the same maladaptive emotion regulation strategies modeled by their mothers. In another cross-sectional study, Buckholdt et al. (2014) reported that adolescents aged 12 to 18 years whose parents expressed higher levels of emotion dysregulation reported previously experiencing more frequent emotional invalidation from their parents, and higher present levels of emotion dysregulation themselves. In a cross-sectional study involving children aged 6 to 12 years old and their married parents, Li et al. (2019) found that both maternal and paternal emotion dysregulation was associated with their children’s emotion dysregulation. Further, they found that a parent’s emotion dysregulation was associated with a decreased ability to appropriately socialize their children’s expression of negative emotion. In regard to the unique role of fathers, a meta-analysis reviewing paternal depression and parenting behavior found that depressed fathers were more likely to display negative parenting behaviors (e.g., hostility) than positive ones (e.g., warmth; Wilson and Durbin, 2010). This suggests that paternal dysfunction likely also plays a role in emotion socialization practices and the transmission of dysfunction to children.

### Transmission of Internalizing Psychopathology and Emotion Dysregulation From Parents to Children

The literature reviewed thus far suggests important links between parent dysfunction (i.e., internalizing psychopathology and emotion dysregulation), supportive and unsupportive emotion socialization, and emotion dysregulation and internalizing psychopathology in children. Although research on subcomponents of this model provides initial support for a broader theoretical model of transmission, no study to date has tested all these paths in tandem. In one cross-sectional study, Suveg et al. (2011) explored the transmission of maternal psychopathology to children aged 7 to 12 years old. The relation between maternal psychopathology and child internalizing and externalizing symptoms was mediated by child emotion regulation. In another cross-sectional study (Kerns et al., 2017) where anxious mothers listened to recordings of distressed children, the authors found that maternal anxiety was positively associated with child anxiety in children aged 3 to 8 years old. This association was sequentially mediated by ineffective maternal emotion regulation and maternal accommodation (e.g., providing excessive reassurance or modifying the environment so that a child can avoid anxiety-provoking situations). In Thomassin et al. (2017), the authors utilized a cross-sectional design to examine the transmission of depressive symptoms from mothers and fathers to children aged 7 to 12 years old. Parental emotion socialization and child emotion regulation mediated the relation between maternal and child symptoms of depression (Thomassin et al., 2017). Notably, the authors did not consider how parent emotion dysregulation is implicated in the transmission of psychopathology from parents to children.

While it is clear that parent dysfunction contributes to child dysfunction, the current study aims to build upon the extant literature by testing a preliminary cascade effect of parent internalizing psychopathology and emotion dysregulation. We propose an emotion socialization transmission framework whereby parent dysfunction is transmitted to children through the impact of supportive and unsupportive emotion socialization on child emotion dysregulation. Specifically, we expected that parent internalizing psychopathology and emotion dysregulation would be associated with higher levels of unsupportive emotion socialization and lower levels of supportive emotion socialization, as has been illustrated in previous literature (e.g., Goodman and Gotlib, 1999; Hoffman et al., 2006; Morris et al., 2007). Consistent with previous findings (e.g., Gottman et al., 1997; Sanders et al., 2015), we expected that higher levels of unsupportive emotion socialization and lower levels of supportive emotion socialization would in turn be associated with higher levels of child emotion dysregulation. We hypothesized that higher levels of child emotion dysregulation would then also be associated with higher levels of child internalizing psychopathology as other researchers have reported (e.g., Silk et al., 2003; Suveg and Zeman, 2004). We expected that this framework would apply to both mothers and fathers, although the specific mechanisms within this framework may differ by parent gender. Gaining a more nuanced understanding about how parent dysfunction is transmitted to children will allow us to further examine specific factors and ways we can support families in order to mitigate negative psychological outcomes for children at risk. We chose to examine this model in middle childhood as it is an important stage of development wherein children are learning about themselves and engaging more with peers, with parents still actively involved in their development.

## Materials and Methods

### Participants

A total of 350 mothers (*M*_age_ = 36.17, *SD* = 7.55) and 355 fathers (*M*_age_ = 35.43, *SD* = 6.49) of children aged 8 to 12 years were recruited from Amazon Mechanical Turk (MTurk^1^) to participate in the current study. All parents were residents of Canada or the United States. Mothers and fathers were not recruited from the same family. The majority of parents identified as White (71%), Black (10.4%), or Latin American (5.5%); reported being married or common law (72%); had at least a university degree (66.7%); and had an income of at least $40,000 (74%).

### Measures

#### Parent Internalizing Psychopathology

Parents completed the Brief Symptoms Inventory-18 (BSI-18; Derogatis, 2001), an 18-item self-report measure of parent psychopathology. Parents were asked to indicate to what extent they are troubled by various symptoms (e.g., “Feeling no interest in things,” “Feeling restless”) on a five-point Likert scale, ranging from 0 “Not at all” to 4 “Very much.” The BSI-18 is comprised of three subscales: Anxiety, Depression, and Somatization, and a global score of all the three subscales. Following previous research, a global score was used for the current study (e.g., Andreu et al., 2008). Good to excellent internal consistency and test–retest reliability for the BSI-18 global score has been established (Derogatis, 2001; Asner-Self et al., 2006). In the current sample, Cronbach’s alpha for mothers and fathers were 0.96 and 0.97, respectively.

#### Parent Emotion Dysregulation

Parents completed the Difficulties in Emotion Regulation Scale (DERS; Gratz and Roemer, 2004), a 36-item self-report questionnaire that assesses problems with emotion regulation. Parents were asked to indicate how often items (e.g., “I have difficulty making sense out of my feelings,” “When I’m upset, I feel out of control”) apply to themselves on a five-point Likert scale, ranging from 1 “Almost never” to 5 “Almost always.” The DERS is comprised of six subscales: (1) Non-acceptance of emotions, (2) Difficulties engaging in goal-directed behavior, (3) Impulse control difficulties, (4) Lack of emotional awareness, (5) Limited access to emotion regulation strategies, (6) Lack of emotional clarity. Following previous research, a total score of all subscales was used for the current study (e.g., Tull et al., 2007). The DERS total score exhibits strong internal consistency (Gratz and Roemer, 2004). In the current sample, Cronbach’s alpha was 0.89 for both mother and father reports.

#### Parental Emotion Socialization

Parents completed the Coping with Children’s Negative Emotions Scale (CCNES; Fabes et al., 2002), a 12-item self-report questionnaire that assesses ways in which parents respond to their children’s expressions of negative emotions. Parents were presented with a series of hypothetical vignettes describing situations in which their child is expressing a negative emotion. For each vignette (e.g., “If my child falls off his/her bike and breaks it, and then gets upset and cries, I would…”), parents were then asked to rate the extent to which they would respond in six distinct ways (i.e., minimizing reactions, punitive reactions, distress reactions, expressive encouragement, problem-focused reactions, emotion-focused reactions). Parents used a seven-point Likert scale, ranging from 1 “Very unlikely” to 7 “Very likely.” Good internal consistency and test–retest reliability, and adequate construct validity have been established for the CCNES (Fabes et al., 2002). Following previous research, the six response types were grouped into two broadband scores: supportive (expressive encouragement, problem-focused, and emotion-focused reactions) and unsupportive (punitive, minimizing, and distress reactions) emotion socialization (e.g., Denham and Kochanoff, 2002). Intercorrelations between supportive subscales ranged from 0.58 to 0.84 and between unsupportive subscales from 0.61 to 0.81. Cronbach’s alphas for supportive emotion socialization were 0.94 for both mothers and fathers. Cronbach’s alphas for unsupportive emotion socialization were 0.83 and 0.88 for mothers and fathers, respectively.

#### Child Emotion Dysregulation

Parents completed the Emotion Regulation Checklist (ERC; Shields and Cicchetti, 1997), a 24-item parent-report measure of child emotion regulation. Parents were asked to rate how often their child exhibits each item on a four-point Likert scale, ranging from 1 “Never” to 4 “Almost always.” The ERC is comprised of two factors: Emotion Regulation (e.g., “Can say when s/he is feeling sad, angry or mad, fearful or afraid”) and Emotion Lability/Negativity (e.g., “Responds negatively to neutral or friendly approaches by peers”). Following previous research, a total dysregulation score which combined both subscales (regulation items were reverse scored prior to combining) was used for the current study (e.g., Ramsden and Hubbard, 2002). Pearson’s correlation between the two subscales was −0.63 for the current sample. The ERC total score exhibits high internal consistency and the ERC has established good convergent validity and good reliability (Shields and Cicchetti, 1997; Ramsden and Hubbard, 2002). Cronbach’s alpha for the current sample was 0.88.

#### Child Internalizing Psychopathology

Parents completed the Brief Problem Monitor (BPM; Achenbach et al., 2001), a 19-item parent-report measure of child psychopathology. Parents were asked to rate each item to describe their child (e.g., “Stubborn, sullen, irritable,” “Unhappy, sad, depressed”) on a three-point Likert scale, ranging from 0 “Not true” to 2 “Very true.” The BPM yields three subscales: Internalizing, Externalizing, and Attention Problems. For the current study, the Internalizing subscale was used. High internal consistency and good test–retest reliability for the BPM have been established (Achenbach et al., 2001; Piper et al., 2014). For the current sample, Cronbach’s alpha was 0.85.

### Procedure

Parents completed the questionnaires on the Qualtrics survey platform via MTurk and received $5 for their participation. After providing informed consent for the study, participants were presented a set of screener questions that participants were required to pass to determine study eligibility. These included questions to assess parent-status and child age, along with others used as filler questions so participants would be unaware of which question was used to assess eligibility (Schleider and Weisz, 2015). Once past the screener, parents were instructed to complete all questions about their child aged 8–12 years. If they had more than one child in this age range, parents were asked to select one and complete all questions about this child. All study procedures were conducted in accordance with the sponsoring university’s research ethics board.

### Data Analytic Plan

**FIGURE 1:**
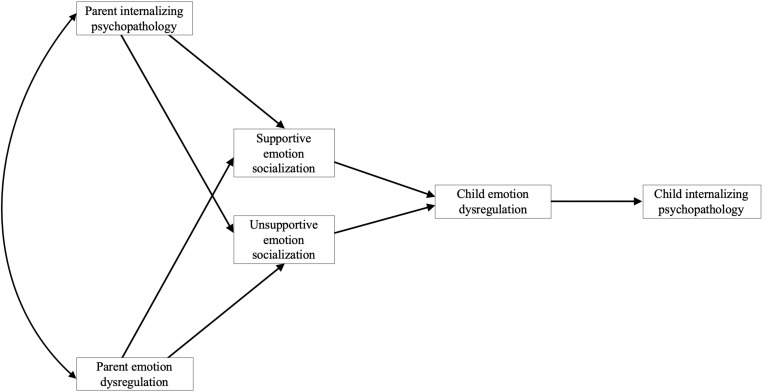
Proposed theoretical model of the transmission of parent internalizing psychopathology and emotion dysregulation to children via supportive and unsupportive emotion socialization and child emotion dysregulation.

We conducted path analyses to examine the validity of our proposed theoretical model as displayed in Figure 1 using Mplus 8.1 (Muthén and Muthén, 1998–2017). The maximum likelihood estimation (MLR estimator) was used, which is robust to non-normality (Muthén and Muthén, 1998–2017). We randomly split our full sample (*N* = 704) in half to create two subsamples: a model development sample (*n* = 352, 51% mothers) and a model validation sample (*n* = 353, 49% mothers). We used the model development sample to develop an empirical model from our initial proposed theoretical model, and the model validation sample to validate the empirical model from the first subsample (i.e., to test whether the final model from our model development approach would be replicated).

#### Model Development

Using the model development sample, we first tested the initial proposed theoretical model (i.e., Figure 1) with parent gender and child age included as covariates in each path of the model, and then used a data-driven approach to guide our addition of specific paths. Paths were added one at a time based on modification indices (MI; cutoff > 10; Muthén and Muthén, 1998–2017), adding the most theoretically sound paths with the largest MI each time (Wang and Wang, 2019). For each model tested, model fit was examined using several fit indices, including the Chi-Square model fit index (cutoff *p* < 0.05), Akaike information criteria (AIC; Akaike, 1974), comparative fit index (CFI; Bentler, 1990; cutoff > 0.95), root mean square error of approximation (RMSEA; Steiger, 1990; cutoff < 0.08), and standardized root mean square residual (SRMR; cutoff < 0.08). If more than one model fit the data well, we conducted a Satorra-Bentler Scaled Chi-Square difference test (Satorra and Bentler, 2010) to test whether the addition of a path (as suggested by the MIs) to the model yielded a significantly better fit than the previous model (i.e., the nested model). Once the best-fitting, most parsimonious model was identified, we then calculated the total, direct, and indirect effects within this model. Then, we tested whether the significant indirect effects differed in size. To do this, we used the Model Constraint command in Mplus to estimate mean differences between the indirect effects, and tested those differences via a series of *z*-tests. Finally, we conducted a multigroup analysis to first calculate the indirect effects in the model for mothers and fathers, and then test whether the significant indirect effects differed in size between mothers and fathers. To do this, we again used the Model Constraint command to estimate differences between each significant indirect effect for mothers versus fathers and tested these differences via *z*-tests.

#### Model Validation

Using the validation sample, we tested the final, best-fitting model as derived from the data-driven approach to determine the validity of the model (i.e., to test whether the final model replicated). Like with the model development sample, we then tested for differences in indirect effects within the model and conducted a multigroup analysis to examine differences in indirect effects for mothers versus fathers.

## Results

### Model Development

#### Descriptive Analyses

Means, standard deviations, ranges, and intercorrelations for all study variables for the model development sample are presented in Table 1. Means, standard deviations, ranges, and intercorrelations for all study variables for this sample split by parent gender are presented in Table 2. As some of the main study variables violated the assumption of normality, maximum likelihood estimation (MLR estimator), which is robust to non-normality, was used in all analyses. Given the bivariate correlations, we examined for possible collinearity between parent internalizing psychopathology and parent emotion dysregulation, and between child internalizing psychopathology and child emotion dysregulation using the variance inflation factors (VIFs) of the coefficients (Tomaschek et al., 2018). All VIF values were below the cutoff of 5 (Sheather, 2009), indicating no concerns with collinearity.

**TABLE 1:** Means (standard deviations), ranges, and intercorrelations among all study variables for the model development sample.

Variable	1	2	3	4	5	6	*M* (*SD*)	Range	
								Min.	Max.
(1) Parent internalizing psychopathology	−						26.28 (12.51)	18.00	88.00
(2) Parent emotion dysregulation	0.65**	−					74.84 (24.77)	36.00	149.00
(3) Supportive emotion socialization	–0.20	−0.34**	−				4.87 (0.97)	1.08	6.67
(4) Unsupportive emotion socialization	0.52**	0.66**	−0.43**	−			2.66 (0.88)	1.00	5.14
(5) Child emotion dysregulation	0.61**	0.65**	−0.37**	0.64**	−		45.45 (7.61)	32.00	66.00
(6) Child internalizing psychopathology	0.63**	0.57**	–0.17	0.51**	0.59**	−	7.95 (2.43)	6.00	18.00

**p < 0.01. Min. = Minimum. Max. = Maximum.

**TABLE 2:** Means (standard deviations) and intercorrelations among all study variables, displayed by mothers and fathers, for the model development sample.

Variable	1	2	3	4	5	6	Mother *M* (*SD*)	Father *M* (*SD*)
(1) Parent internalizing psychopathology	−	0.57**	–0.13	0.40**	0.52**	0.52**	24.86 (10.63)	27.72 (14.06)
(2) Parent emotion dysregulation	0.73**	−	−0.36**	0.61**	0.59**	0.44**	74.81 (25.22)	75.90 (24.32)
(3) Supportive emotion socialization	−0.22**	−0.31**	−	−0.50**	−0.42**	–0.08	5.07 (0.90)	4.67 (0.99)
(4) Unsupportive emotion socialization	0.60**	0.70**	−0.36**	−	0.60**	0.43**	2.59 (0.83)	2.73 (0.93)
(5) Child emotion dysregulation	0.68**	0.72**	−0.29**	0.67**	−	0.59**	44.41 (7.37)	46.52 (7.72)
(6) Child internalizing psychopathology	0.72**	0.69**	−0.22**	0.55**	0.68**	−	7.80 (2.36)	8.10 (2.50)

Correlations for mothers (*N* = 178) appear below the diagonal and correlations for fathers (*N* = 174) appear above the diagonal. **p < 0.01.

#### Model Testing

**FIGURE 2:**
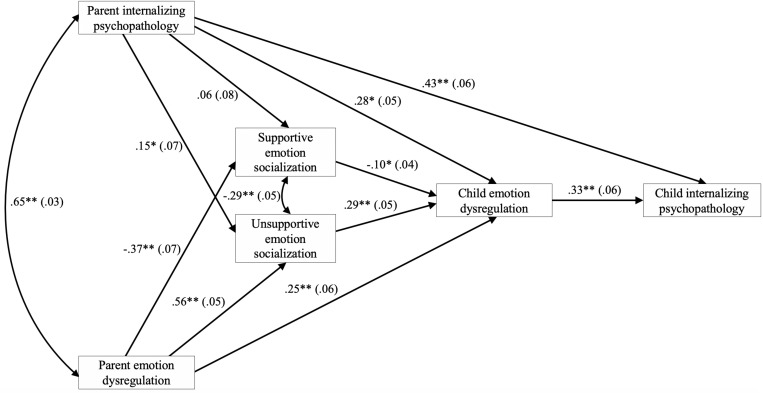
Final, best-fitting model, within the model development sample, of the transmission of parent internalizing psychopathology and emotion dysregulation to children’s internalizing psychopathology as sequentially mediated by parental emotion socialization and child emotion dysregulation. Path coefficients are standardized. Values in parentheses are standard errors. *p < 0.05. **p ≤ 0.001.

Our initial, most parsimonious theoretical model (Model 1; see Figure 1) did not provide a good fit to the data (see Table 3 for fit statistics). Modification indices recommended adding a direct path from parent internalizing psychopathology to child emotion dysregulation (Model 2). Adding this path improved fit but recommended model fit indices were still not met. Modification indices also recommended adding a direct path from parent to child internalizing psychopathology (Model 3). Again, the addition of this path improved fit but not sufficiently. Modification indices then recommended adding a correlation between supportive and unsupportive emotion socialization (Model 4). Once more, the addition of this path improved fit but still not sufficiently. Finally, modification indices recommended adding a direct path between parent and child emotion dysregulation (Model 5). With this addition, the model fit the data well and there were no further paths suggested by modification indices. The Satorra-Bentler Scaled Chi-Square difference test indicated that Model 5 was a significantly better model fit than Model 4, χ^2^(1) = 14.68, *p* < 0.001. We therefore retained Model 5 for our analysis of indirect effects. The final, best-fitting model is displayed in Figure 2. Parent gender and child age were included as covariates in each path of the model. Child age was not a significant covariate. Parent gender was a significant covariate for the path including supportive emotion socialization only, whereby mothers were more likely to report supportive emotion socialization practices than fathers, β = −0.21, 95% CI [−0.30, −0.11]. *R*^2^ values in the model were significant at *p* < 0.001 for supportive emotion socialization, *R*^2^ = 0.151, unsupportive emotion socialization, *R*^2^ = 0.447, child emotion dysregulation, *R*^2^ = 0.404, and child internalizing psychopathology, *R*^2^ = 0.346.

**TABLE 3:** Summary of model fit statistics for all path analytic models tested using the model development sample.

Model tested	Chi-Square test of model fit	AIC	CFI	RMSEA	SRMR
Model 1: Proposed theoretical model	χ^2^(11, *N* = 352) = 166.08, *p* < 0.001	11209.06	0.74	0.20	0.12
Model 2: Direct path from parent internalizing psychopathology to child emotion dysregulation added	χ^2^(10, *N* = 352) = 107.80, *p* < 0.001	11139.68	0.84	0.17	0.08
Model 3: Direct path from parent internalizing psychopathology to child internalizing psychopathology added	χ^2^(9, *N* = 352) = 62.44, *p* < 0.001	11072.74	0.91	0.13	0.06
Model 4: Correlation between supportive and unsupportive emotion socialization added	χ^2^(8, *N* = 352) = 36.89, *p* < 0.001	11043.06	0.95	0.10	0.04
**Model 5: Direct path from parent to child emotion dysregulation added**	**χ^2^(7, *N* = 352) = 19.77, *p* = 0.006**	**11025.24**	**0.98**	**0.07**	**0.03**

*The “Model tested” column indicates the new addition to the previous model that comprises each new model tested. Model in bold is the best-fitting model. AIC = Akaike information criteria. CFI = comparative fit index. RMSEA = root mean square error of approximation. SRMR = standardized root mean square residual.*

#### Test of Indirect Effects

Based on the final model displayed in Figure 2, we tested six indirect paths (see Table 4). The indirect paths from both parent internalizing psychopathology *and* parent emotion dysregulation to child internalizing psychopathology through unsupportive emotion socialization and child emotion dysregulation were significant, but the indirect paths through supportive emotion socialization were not. Additionally, the indirect paths from both parent internalizing psychopathology *and* parent emotion dysregulation to child internalizing psychopathology through child emotion dysregulation alone were significant.

**TABLE 4:** Summary of direct effect, total effects, and indirect effects tested with the final model for the model development sample.

	Effects	β	*SE*	95% CI
	Total effect from parent to child internalizing psychopathology	0.533	0.057	[0.420, 0.646]
	Direct effect from parent to child internalizing psychopathology	0.429	0.064	[0.304, 0.554]
	Total effect from parent emotion dysregulation to child internalizing psychopathology	0.147	0.034	[0.080, 0.215]
	Indirect effects:			
(1)	Parent to child internalizing psychopathology via unsupportive emotion socialization and child emotion dysregulation	0.014	0.006	[0.002, 0.026]
(2)	Parent to child internalizing psychopathology via supportive emotion socialization and child emotion dysregulation	−0.002	0.003	[−0.008, 0.003]
(3)	Parent emotion dysregulation to child internalizing psychopathology via unsupportive emotion socialization and child emotion dysregulation	0.053	0.017	[0.020, 0.087]
(4)	Parent emotion dysregulation to child internalizing psychopathology via supportive emotion socialization and child emotion dysregulation	0.012	0.006	[0.000, 0.023]
(5)	Parent to child internalizing psychopathology via child emotion dysregulation	0.092	0.022	[0.049, 0.135]
(6)	Parent emotion dysregulation to child internalizing psychopathology via child emotion dysregulation	0.082	0.024	[0.034, 0.130]

*β = standardized beta. *SE* = standard error. 95% CI = 95% confidence intervals around β.*

Given these four significant indirect effects, we tested whether these effects differed significantly from each other. Results from *z*-tests indicated that the indirect effect from parent to child internalizing psychopathology through child emotion dysregulation alone was significantly larger than the indirect effects from parent internalizing psychopathology and parent emotion dysregulation to child internalizing psychopathology through unsupportive emotion socialization and child emotion dysregulation, *z* = −0.02, 95% CI [−0.02, −0.01], and *z* = −0.01, 95% CI [−0.02, −0.01], respectively. No other significant indirect effects significantly differed from each other.

We conducted a multigroup analysis by parent gender, followed by *z*-tests, to test whether any of the four significant indirect effects differed for mothers versus fathers and they did not, *z* = −0.00, 95% CI [−0.01, 0.00], *z* = −0.00, 95% CI [−0.01, 0.00], *z* = 0.00, 95% CI [−0.02, 0.01], and *z* = −0.01, 95% CI [−0.02, 0.00], for the first, third, fifth, and sixth indirect effects (see Table 4), respectively.

### Model Validation

#### Descriptive Analyses

Means, standard deviations, ranges, and intercorrelations for all study variables for the model validation sample as a whole are presented in Table 5. Means, standard deviations, ranges, and intercorrelations for all study variables for this sample are presented in Table 6. As some of the main study variables violated the assumption of normality, maximum likelihood estimation (MLR estimator), which is robust to non-normality, was used in all analyses. Similar to the model development sample, we examined for possible collinearity for parent internalizing psychopathology and parent emotion dysregulation, and for child internalizing psychopathology and child emotion dysregulation using the VIFs of the coefficients (Tomaschek et al., 2018). All VIF values were below the cutoff of 5 (Sheather, 2009), indicating no concerns with collinearity.

**TABLE 5:** Means (standard deviations), ranges, and intercorrelations among all study variables for the model validation sample.

Variable	1	2	3	4	5	6	*M* (*SD*)	Range	
								Min.	Max.
(1) Parent internalizing psychopathology	−						26.56 (13.00)	18.00	84.00
(2) Parent emotion dysregulation	0.61**	−					75.73 (25.05)	36.00	178.00
(3) Supportive emotion socialization	−0.21**	−0.37**	−				4.88 (0.90)	2.08	7.00
(4) Unsupportive emotion socialization	0.49**	0.64**	−0.43**	−			2.68 (0.90)	1.00	5.03
(5) Child emotion dysregulation	0.50**	0.58**	−0.25**	0.61**	−		45.59 (7.47)	33.00	72.00
(6) Child internalizing psychopathology	0.62**	0.48**	−0.12	0.40**	0.54**	−	8.10 (2.65)	6.00	18.00

**p < 0.01. Min. = Minimum. Max. = Maximum.

**TABLE 6:** Means (standard deviations) and intercorrelations among all study variables, displayed by mothers and fathers, for the model validation sample.

Variable	1	2	3	4	5	6	Mother *M* (*SD*)	Father *M* (*SD*)
(1) Parent internalizing psychopathology	−	0.61**	−0.21**	0.43**	0.49**	0.57**	26.97 (13.04)	26.16 (12.99)
(2) Parent emotion dysregulation	0.62**	−	−0.44**	0.57**	0.56**	0.45**	75.01 (25.71)	76.41 (24.47)
(3) Supportive emotion socialization	−0.24**	−0.30**	−	−0.51**	−0.31**	−0.07	5.06 (0.90)	4.71 (0.86)
(4) Unsupportive emotion socialization	0.54**	0.70**	−0.37**	−	0.61**	0.40**	2.67 (0.87)	2.69 (0.92)
(5) Child emotion dysregulation	0.52**	0.60**	−0.18*	0.61**	−	0.59**	45.27 (7.61)	45.90 (7.34)
(6) Child internalizing psychopathology	0.66**	0.52**	−0.18*	0.41**	0.51**	−	8.14 (2.71)	8.07 (2.60)

Correlations for mothers (*N* = 172) appear below the diagonal and correlations for fathers (*N* = 181) appear above the diagonal. *p < 0.05. **p < 0.01.

#### Model Testing

**FIGURE 3:**
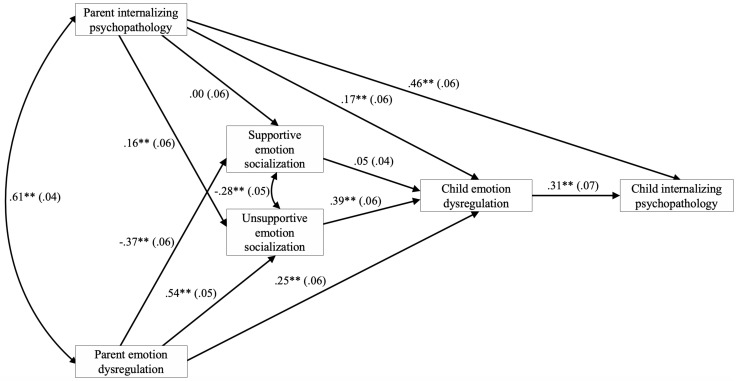
Final, best-fitting model, within the model validation sample, of the transmission of parent internalizing psychopathology and emotion dysregulation to children’s internalizing psychopathology as sequentially mediated by parental emotion socialization and child emotion dysregulation. Path coefficients are standardized. Values in parentheses are standard errors. **p ≤ 0.001.

The goal of the model testing for the model validation sample was to test whether the final, best-fitting model from our model development approach could be replicated in a second sample. The final model derived from the data-driven approach in the model development sample demonstrated excellent overall model fit in the model validation sample, χ^2^(8, *N* = 353) = 4.98, *p* = 0.662, AIC = 11231.07, CFI = 1.00, RMSEA = 0.00, SRMR = 0.01, demonstrating validity for the final model. This model, along with path coefficients and standard errors, are displayed in Figure 3. Thus, we proceeded to calculate and test indirect effects. Parent gender and child age were included as covariates in each path of the model. Like in the model development sample, child age was not a significant covariate. Also akin to the model development sample, parent gender was a significant covariate for the path including supportive emotion socialization only, whereby mothers were more likely to report supportive emotion socialization practices than fathers, β = −0.18, 95% CI [−0.28, −0.09]. *R*^2^ values in the model were significant at *p* < 0.001 for supportive emotion socialization, *R*^2^ = 0.161, unsupportive emotion socialization, *R*^2^ = 0.420, child emotion dysregulation, *R*^2^ = 0.456, and child internalizing psychopathology, *R*^2^ = 0.448.

#### Test of Indirect Effects

Based on the final model from the model development sample, we tested six indirect paths (see Table 7). The results replicated those from the model development sample. The indirect paths from both parent internalizing psychopathology *and* parent emotion dysregulation to child internalizing psychopathology through unsupportive emotion socialization and child emotion dysregulation were significant, but the indirect paths through supportive emotion socialization were not. Additionally, the indirect paths from both parent internalizing psychopathology *and* parent emotion dysregulation to child internalizing psychopathology through child emotion dysregulation alone were significant. Given these four significant indirect effects, we tested whether these effects differed significantly from each other. Results from *z*-tests indicated that no significant indirect effect significantly differed from another.

**TABLE 7:** Summary of direct effect, total effects, and indirect effects tested with the final model of the model validation sample.

	Effects	β	*SE*	95% CI
	Total effect from parent to child internalizing psychopathology	0.533	0.056	[0.423, 0.644]
	Direct effect from parent to child internalizing psychopathology	0.461	0.062	[0.338, 0.583]
	Total effect from parent emotion dysregulation to child internalizing psychopathology	0.133	0.037	[0.062, 0.205]
	Indirect effects:			
(1)	Parent to child internalizing psychopathology via unsupportive emotion socialization and child emotion dysregulation	0.019	0.008	[0.003, 0.035]
(2)	Parent to child internalizing psychopathology via supportive emotion socialization and child emotion dysregulation	0.000	0.001	[−0.002, 0.002]
(3)	Parent emotion dysregulation to child internalizing psychopathology via unsupportive emotion socialization and child emotion dysregulation	0.063	0.019	[0.027, 0.100]
(4)	Parent emotion dysregulation to child internalizing psychopathology via supportive emotion socialization and child emotion dysregulation	−0.006	0.006	[−0.018, 0.006]
(5)	Parent to child internalizing psychopathology via child emotion dysregulation	0.053	0.021	[0.013, 0.094]
(6)	Parent emotion dysregulation to child internalizing psychopathology via child emotion dysregulation	0.076	0.028	[0.021, 0.132]

*β = standardized beta. *SE* = standard error. 95% CI = 95% confidence intervals around β.*

We conducted a multigroup analysis by parent gender followed by *z*-tests in order to test whether any of the four significant indirect effects differed for mothers versus fathers and they did not, *z* = 0.01, 95% CI [−0.00, 0.02], *z* = 0.00, 95% CI [−0.00, 0.01], *z* = 0.01, 95% CI [−0.01, 0.02], and *z* = 0.00, 95% CI [−0.01, 0.02] for the first, third, fifth, and sixth indirect effects (see Table 7), respectively.

## Discussion

Researchers have indicated that children of parents with dysfunction are at an increased risk of dysfunction themselves (Goodman and Brumley, 1990; Lapalme et al., 1997; DelBello and Geller, 2001; Buckholdt et al., 2014). Current evidence suggests that parental emotion socialization may play an important role in the transmission of parent dysfunction from parent to child (Suveg et al., 2011; Kerns et al., 2017; Thomassin et al., 2017). The present study contributes new insights to current models of how internalizing psychopathology and emotion dysregulation transmit from parent to child. Study findings provide preliminary support for a cascade effect of parent dysfunction whereby both parent internalizing psychopathology and emotion dysregulation are associated with unsupportive emotion socialization practices, which are in turn related to increased child emotion dysregulation and higher levels of child internalizing symptoms. As expected, these indirect effects held for both mothers and fathers and across children aged 8–12 years.

Indirect effects in the model indicated that parent dysfunction is associated with increased levels of unsupportive emotion socialization, which are then associated with negative consequences for children’s emotion regulation and subsequent development of internalizing psychopathology. Given that current evidence indicates that internalizing psychopathology and emotion dysregulation are moderately heritable (Rutter et al., 1999a,b; Hawn et al., 2015; McRae et al., 2017), children might inherit from their parents underlying tendencies that predispose them to this dysfunction. These innate risk factors may then interact with the compromised emotion socialization that parents with dysfunction engage in. For instance, children may learn from watching their parents use maladaptive emotion regulation strategies while dealing with internalizing disorders, and from negative parental reactions to their own expressions of emotion. This may leave children without strategies for effectively regulating their own emotions and, as a result, position them at elevated risk for internalizing psychopathology (Bandura, 1986; Moffitt et al., 2006; Rutter et al., 2006).

Importantly, although the model included a correlation between unsupportive and supportive emotion socialization—indicating a relationship between the two—the indirect paths from parent internalizing psychopathology and emotion dysregulation to children that included supportive emotion socialization were *not* significant. This suggests potential distinct influences of these two categories of parental emotion socialization. Our findings indicate that, in the transmission of parent dysfunction to children, increased unsupportive emotion socialization is more impactful than decreased supportive emotion socialization. This is not too surprising given that researchers have viewed these two categories of emotion socialization as independent constructs that can co-occur in parent–child interactions but may differentially operate on children’s development and outcomes (Lunkenheimer et al., 2007; Miller-Slough et al., 2016; Ramakrishnan et al., 2019). Indeed, researchers previously examining the role of emotion socialization on child emotion regulation also found an effect of unsupportive emotion socialization with no effect of supportive emotion socialization (Williams and Woodruff-Borden, 2015). This pattern of findings suggests that targeting the reduction of unsupportive emotion socialization practices may be a more promising means to disrupt the transmission of dysfunction from parent to child than aiming to increase supportive emotion socialization practices. However, in order to further elucidate the role of emotion socialization in this context, it is recommended that future researchers examine the path of transmission of dysfunction from parent to child via emotion parenting with multiple measures of parental emotion socialization. Such measures might assess parents’ teaching and modeling of emotion skills for their children, in addition to parent reactions to children’s emotion expressions, more directly (Gottman et al., 1996, 1997; Eisenberg et al., 1998).

In the model development sample and the validation sample, there were significant indirect effects from parent emotion dysregulation to child internalizing psychopathology through emotion dysregulation alone. Notably, these did not include parental emotion socialization. Further, for the model development sample, the indirect effect from parent to child internalizing psychopathology through child emotion dysregulation alone was larger than the indirect effect through unsupportive emotion socialization. However, this difference in size between indirect effects was not found in the model validation sample, suggesting it may have been a spurious result. Nevertheless, these effects suggest that, although unsupportive emotion socialization may be one mechanism by which parent dysfunction is transmitted to children, there are other factors not accounted for in the final model that may contribute to an equal or larger degree (e.g., parenting stress, other dimensions of parenting), which warrant further investigation in this context. Additionally, the possibility of a spurious result as described suggests that further replication studies examining this transmission model are necessary.

Lastly, the final model and the four significant indirect effects were applicable to both mothers and fathers and were consistent across children within the developmental period spanning ages 8 through 12 (i.e., middle childhood). Differences between maternal and paternal associations to child emotion regulation in children in middle childhood have been noted in previous literature, however. For example, in children aged 7–12 years, Thomassin et al. (2017) found that parental positive affect and child emotion dysregulation mediated the relation between parent and child symptoms of depression, but only for mothers. However, our study findings related to parental negative emotion (i.e., unsupportive socialization; punitive, distressful responses) in relation to child emotion regulation, which may explain why these findings differ. More specifically, literature has suggested that mothers show more positive affect toward their children than fathers (Cassidy et al., 1992; Garner et al., 1997), consistent with gender stereotypes delineating greater emotionality in women than men (Fabes and Martin, 1991), which may have contributed to Thomassin et al.’s (2017) finding. On the other hand, fathers’ tendency to display more negative emotion in socialization (compared to positive; Cassano et al., 2007; Engle and McElwain, 2010), along with mothers’ greater emotionality overall (including negative emotion; Brody, 2000; Wong et al., 2009) may diminish parent gender differences regarding parental negative emotion. Our results also coincided with findings by Li et al. (2019), whereby parent emotion dysregulation and socialization of negative emotion were associated with child emotion dysregulation for both mothers and fathers of children aged 6 to 12 years. Additional examination of the transmission of parent dysfunction to children would be beneficial to further understand different parent effects. Even so, within the complex etiological processes at work in the development of child dysfunction, the present work provides some initial support for a mechanism by which maternal and paternal internalizing psychopathology and emotion dysregulation disrupt parental emotion socialization by increasing unsupportive emotion socialization practices, which then increase child emotion dysregulation and risk for child internalizing symptoms.

This study is not without limitations. We are unable to make causal claims given that the data collected are cross-sectional. Our sample was also rather homogenous and gathered from MTurk which limits the generalizability of our findings. The study used self-report and parent-report questionnaires to measure parents’ emotion socialization practices, and parent and child emotion regulation and internalizing psychopathology, which present methodological limitations. For one, self-report measures are susceptible to social desirability bias (e.g., Kendziora and O’Leary, 1998). Additionally, our findings may have been confounded by the presence of same-reporter bias and common-method variance in data collection. Future research would benefit from a focus on longitudinal designs with more heterogeneous samples and additional methods, such as laboratory observation and the use of multiple informants. Nonetheless, a longitudinal design testing sequential mediation would require multiple time points, and thus a preliminary study such as this one offers confidence that such a study would be worthwhile to conduct. It would also be prudent to investigate whether the present model is also applicable to externalizing psychopathology. Additionally, in light of current evidence suggesting that what constitutes “supportive” and “unsupportive” emotion socialization is culturally embedded and varies significantly across child ages, it would also be important to examine the generalizability of our model to younger children and adolescents, and across cultures (Mirabile et al., 2018; Raval et al., 2018). Finally, although it is well-understood that parents influence their children, researchers have shown that children also influence their parents’ behavior (e.g., Cho et al., 2016; Perry et al., 2018), which was not accounted for in our transmission model and warrants investigation in this context.

## Conclusion

Our current findings provide support for a preliminary model of the transmission of internalizing psychopathology and emotion dysregulation from parent to child using a parental emotion socialization framework. Specifically, findings contribute evidence that higher levels of unsupportive emotion socialization practices, but not lower levels of supportive emotion socialization practices, mediate the relation between parent and child dysfunction, which adds to the body of literature examining supportive and unsupportive emotion socialization as distinct factors (e.g., Williams and Woodruff-Borden, 2015; Miller-Slough et al., 2016). Importantly, these findings hold implications for disrupting the transmission of dysfunction and promoting healthy psychological outcomes for at-risk children of parents with dysfunction. Our results suggest that teaching parents with dysfunction *not* to engage in unsupportive emotion socialization practices may be a promising way to deter the transmission of dysfunction.

## Data Availability Statement

The data analyzed in this study is subject to the following licenses/restrictions: The datasets analyzed for this study are available on request to the corresponding author, JAS, jseddon@uoguelph.ca.

## Ethics Statement

The studies involving human participants were reviewed and approved by the Research Ethics Board at the University of Guelph. The participants provided their written informed consent to participate in this study.

## Author Contributions

JAS conceptualized the study and analyzed and interpreted the data. KT made substantial contributions to the interpretation of data and reviewed and revised the manuscript critically. All authors wrote, revised, and approved the final manuscript.

## Conflict of Interest

The authors declare that the research was conducted in the absence of any commercial or financial relationships that could be construed as a potential conflict of interest.

## Footnotes

1 https://www.mturk.com
